# Leveraging Bioinformatics and Machine Learning for Identifying Prognostic Biomarkers and Predicting Clinical Outcomes in Lung Adenocarcinoma

**DOI:** 10.3390/genes15121497

**Published:** 2024-11-21

**Authors:** Kaida Cai, Wenzhi Fu, Hanwen Liu, Xiaofang Yang, Zhengyan Wang, Xin Zhao

**Affiliations:** 1 Department of Epidemiology and Biostatistics, School of Public Health, Southeast University, Nanjing 210009, China; 2 Department of Statistics and Actuarial Science, School of Mathematics, Southeast University, Nanjing 211189, China; 220241993@seu.edu.cn (W.F.); 220242066@seu.edu.cn (H.L.); xiaofangyang@seu.edu.cn (X.Y.); zhengyanwang@seu.edu.cn (Z.W.); xinzhaomath@seu.edu.cn (X.Z.); 3 Key Laboratory of Environmental Medicine Engineering, Ministry of Education, School of Public Health, Southeast University, Nanjing 210009, China; 4 Key Laboratory of Measurement and Control of Complex Systems of Engineering, Ministry of Education, Southeast University, Nanjing 210096, China

**Keywords:** lung adenocarcinoma, RNA sequencing data, machine learning, feature selection, prognostic biomarkers

## Abstract

**Background/Objectives:** There exist significant challenges for lung adenocarcinoma (LUAD) due to its poor prognosis and limited treatment options, particularly in the advanced stages. It is crucial to identify genetic biomarkers for improving outcome predictions and guiding personalized therapies. **Methods:** In this study, we utilize a multi-step approach that combines principled sure independence screening, penalized regression methods and information gain to identify the key genetic features of the ultra-high dimensional RNA-sequencing data from LUAD patients. We then evaluate three methods of survival analysis: the Cox model, survival tree, and random survival forests (RSFs), to compare their predictive performance. Additionally, a protein–protein interaction network is used to explore the biological significance of identified genes. **Results:** *DKK1* and *TNS4* are consistently selected as significant predictors across all feature selection methods. The Kaplan–Meier method shows that high expression levels of these genes are strongly correlated with poorer survival outcomes, suggesting their potential as prognostic biomarkers. RSF outperforms Cox and survival tree methods, showing higher AUC and C-index values. The protein–protein interaction network highlights key nodes such as *VEGFC* and *LAMA3*, which play central roles in LUAD progression. **Conclusions:** Our findings provide valuable insights into the genetic mechanisms of LUAD. These results contribute to the development of more accurate prognostic tools and personalized treatment strategies for LUAD.

## 1. Introduction

Despite advances in early detection and targeted therapies, the outlook for LUAD remains bleak, with high mortality rates largely attributed to late-stage diagnoses and restricted treatment options [1,2]. Current therapeutic approaches, including chemotherapy, immunotherapy, and targeted treatments like EGFR and ALK inhibitors, provide modest survival benefits; however, they fall short in managing the disease over the long term for most patients [3,4,5]. This highlights an urgent need for more effective prognostic markers and personalized treatment strategies to improve patient outcomes in LUAD.

The genetic landscape of LUAD is highly complex, with numerous genes implicated in its initiation, progression, and therapeutic response. Identifying key genetic markers that are strongly associated with LUAD survival requires robust feature selection techniques, especially when analyzing the RNA sequencing data with ultra-high dimension, where the number of genes is greater than the number of samples [6]. High-dimensional data poses challenges to statistical methods due to the risk of overfitting and increased computational complexity, necessitating the development of specialized strategies to manage and interpret such data [7]. In this context, feature selection plays a crucial role in reducing dimensionality, thereby enhancing the interpretability and predictive power of survival models. One approach to tackling the dimensionality challenge in ultra-high dimensional datasets is the use of principled sure independence screening (PSIS), a technique that helps filter out irrelevant variables, leaving a more manageable number of predictors for survival analysis [8]. Following this initial reduction, penalized regression techniques like the smoothly clipped absolute deviation (SCAD) and least absolute shrinkage and selection operator (Lasso) are widely used to enhance the selection process, focusing on retaining only the most relevant features [9,10]. These methods have proven effective in dealing with the multicollinearity and sparsity issues inherent in genetic data. Additionally, the incorporation of information-theoretic concepts like information gain (IG) can further enhance the feature selection process by assessing the importance of each feature in connection to patient survival [11].

Recently, the machine learning approaches have proven invaluable in analyzing high-dimensional biological data, where they enable the discovery of intricate patterns that might otherwise remain hidden [12,13]. Machine learning approaches have been effectively applied to identify key cancer biomarkers, predict disease progression, and develop customized treatment strategies. For instance, support vector machines (SVMs) were employed to differentiate cancer types using gene expression data, achieving high prediction accuracy that supports clinical decision-making [14]. Beyond these applications, machine learning approaches have also emerged as powerful tools in survival analysis due to their ability to handle complex, nonlinear relationships in data without relying on strict parametric assumptions [15]. For instance, the random survival forest (RSF) enhances the capabilities of random forests to survival data, offering a robust, nonparametric approach that can capture interactions between variables and accommodate censoring in survival data [16,17]. Unlike the Cox model, RSF is entirely data-driven and adapts to the underlying structure of the data [16,17,18]. This flexibility makes RSF particularly well-suited for high-dimensional datasets, such as those generated by high-throughput genomic technologies.

In recent years, studies leveraging next-generation sequencing (NGS) data have increasingly applied statistical and machine learning approaches to identify key genetic features linked to survival outcomes in LUAD patients, highlighting the need for effective feature selection strategies to handle the high dimensionality of such datasets [19,20,21]. Traditional feature selection techniques often struggle with genomic data with ultra-high dimensions, underscoring the importance of robust techniques to filter out irrelevant variables [6,7]. Our study addresses this challenge by employing a comprehensive approach that integrates principled sure independence screening (PSIS) for initial dimensionality reduction, followed by penalized regression techniques like Lasso, SCAD and information gain-based methods, to refine the selection of relevant genetic markers [8,9,10]. In contrast to many black-box machine learning techniques, our feature selection approach maintains statistical interpretability, allowing for a clearer understanding of the relationships between selected genetic markers and their impact on survival outcomes. To assess the predictive power of these markers, we conduct a comparative analysis using three methods for survival analysis: the Cox model, survival tree, and random survival forests [16,17,18,22]. This integrated methodology not only enhances the identification of key genetic markers, but also provides a thorough evaluation of survival analysis methods tailored to ultra-high dimensional LUAD data, contributing to more accurate prognostic models and personalized treatment strategies.

## 2. Materials and Methods

### 2.1. Data Extraction and Processing

This study examines RNA sequencing data and clinical information for lung adenocarcinoma (LUAD), obtained from The Cancer Genome Atlas (TCGA) through the Genomic Data Commons (GDC) portal. Initially, the dataset includes 585 samples covering 60,616 gene expression variables. To ensure consistency in survival analysis, 61 samples not classified as either primary tumors or normal solid tissues are systematically removed, along with 11 duplicate entries. Additionally, in line with best practices for survival analysis, we eliminate samples that either lack survival information or have a recorded survival duration of zero. Gene features with zero expression for all samples are also excluded to improve data quality. Following these rigorous screening steps, the resulting dataset consists of 500 samples, encompassing 57,732 gene features.

**Figure 1:**
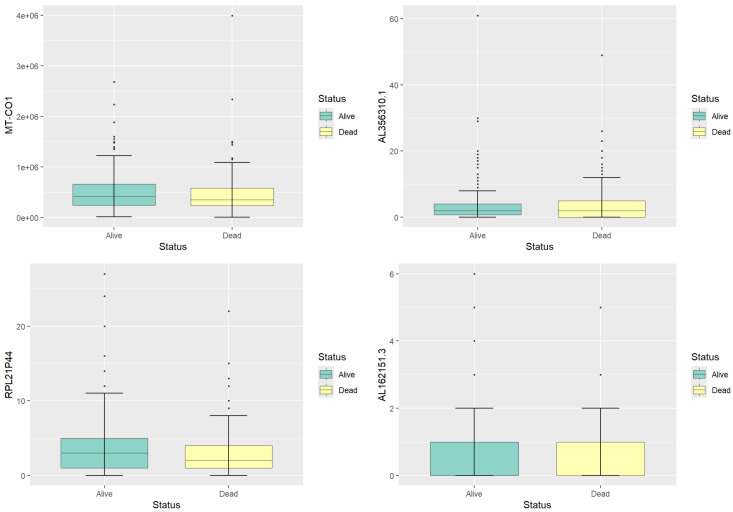
Boxplots illustrating expression levels for the four selected genes.

Figure 1 and Table 1 present boxplots and a summary table for four selected genes (*MT-CO1*, *AL356310.1*, *RPL21P44*, and *AL162151.3*) to visualize the expression patterns. These genes are chosen to reflect a range of expression levels, providing insight into the distribution of gene expression within the dataset. The summary statistics show that MT-CO1 has the highest median expression among the four genes, with a broad distribution range across both survival groups. In contrast, *AL162151.3* displays low expression levels, with many samples showing near-zero values. *AL356310.1* and *RPL21P44* exhibit moderate expression levels, with their medians and means remaining relatively close, suggesting a consistent expression pattern. The comparative analysis of these gene expression levels indicates no significant differences between the ‘Alive’ and ‘Dead’ groups. Therefore, further feature selection is required to select genes that may have significant prognostic implications.

**Figure 2:**
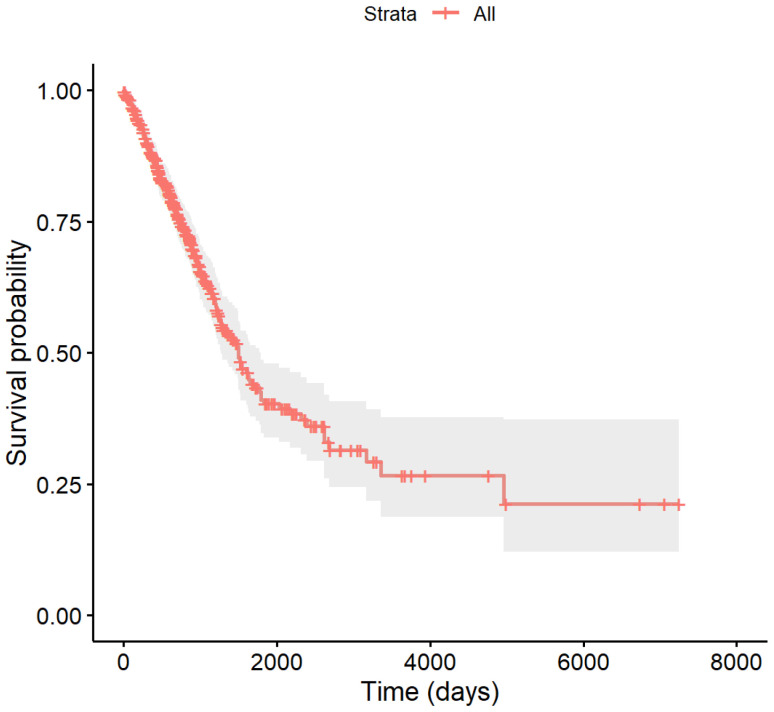
The overall survival curve of 500 LUAD samples.

The follow-up period for the LUAD samples spans from 0.01 to 19.86 years, during which 320 patients succumbed to the illness, leading to a censoring rate of 64%. As depicted in the survival curve in Figure 2, the initial survival probability is 100%, but shows a significant decline within the first five years, ultimately stabilizing around 41.33% at the end of this time frame. The downward steps in the curve correspond to death events, while the cross marks represent censored observations. The gray shading highlights the 95% confidence interval surrounding the survival estimate.

Due to the ultra-high dimensionality, where the number of gene expression features far exceeds the number of samples, it is essential to employ specialized techniques for analysis. RNA sequencing data variability often stems from differences in sequencing depth and sample composition, which may introduce inconsistencies in comparisons. To mitigate these issues, we utilize the Trimmed Mean of M-values normalization approach, to standardize expression levels across samples, thus enhancing the reliability of subsequent analyses [23]. Our study integrates a feature selection strategy that combines principled sure independence screening (PSIS) with the Cox model (Cox), as well as penalized regression techniques like the Lasso and SCAD. Additionally, we apply the information gain (IG) method to identify influential features associated with survival outcomes. Following this feature selection phase, we evaluate the performance of three survival analysis methods: Cox models, survival trees (ST), and random survival forest (RSF), to examine their ability to predict survival based on the selected features. This comprehensive approach enables a comparative analysis of the effectiveness of each method, guiding us toward the optimal strategy for modeling survival data.

### 2.2. Cox Proportional Hazards Model

The Cox model is a semi-parametric method widely used in survival analysis. As described by Lawless [18], the hazard function at any point in time *t*, with respect to a set of covariates $X=(X_{1},X_{2},\ldots ,X_{p})$, can be represented by

(1) $$h\left(t,X\right)=h_{0}\left(t\right)\exp\left(X^{T}\beta \right),$$

where $h_{0}\left(t\right)$ denotes the baseline hazard function, which corresponds to the hazard when all covariates *X* are zero. The factor $\exp(X^{T}\beta )$ adjusts the baseline hazard, capturing the influence of the covariates on the risk level.

In this framework, *C* denotes the censoring time, while Y=min{t,C} represents the observed time, which could correspond to either an event or a censoring point. The indicator variable δ=I(t≤C) indicates whether the event has occurred (δ=1) or if the data are censored (δ=0). Assuming conditional independence of *X* and *Y* given *C*, we work with observed data comprising independent and identically distributed samples $\left\{\left(x_{i},y_{i},\delta_{i}\right):x_{i}\in R^{p},y_{i}\in R^{+},\delta_{i}\in \left\{0,1\right\},i=1,2,\ldots ,n\right\}$. The risk set at time *t*, denoted as R(t), includes all individuals who are still at risk at time *t*, specifically those for whom $y_{i}\geq t$. The partial likelihood function used for estimating the regression coefficients β is expressed as

(2) $$\ell \left(\beta \right)=\sum_{i=1}^{n}\delta_{i}x_{i}^{T}\beta -\sum_{i=1}^{n}\delta_{i}\ln\left\{\sum_{j\in R(y_{i})}\exp\left(x_{j}^{T}\beta \right)\right\}.$$

### 2.3. Feature Selection

Our analysis of survival outcomes involves dealing with ultra-high dimensional datasets, where the number of features (*p*) greatly surpasses the number of observations (*n*). This scenario (p>n) poses significant challenges, including risks of overfitting, computational inefficiencies, and difficulties in model interpretation [6]. To tackle these issues, we implement the principled sure independence screening technique, which effectively decreases the dimensionality from p>n to a more manageable scale where p<n [8]. After this initial screening step, we further narrow down the selection of relevant features using penalized methods, such as the Lasso and the SCAD, along with the information gain method, tailored specifically for the Cox model. This multi-step approach enables us to retain the most influential predictors of survival outcomes, thereby improving both the clarity and effectiveness of our methods.

#### 2.3.1. Principled Sure Independence Screening

Principled sure independence screening for Cox model, introduced by Zhao and Li [8], is a method designed to manage feature selection in ultra-high dimensional data. A central element of this approach is the selection of the parameter $\gamma_{n}$, which regulates the false positive rate, denoted as $q_{n}$. The implementation of PSIS follows a three-step process. In the first step, marginal Cox models are fitted to each individual covariate, yielding estimates for the parameters ${\hat{\beta }}_{j}$ and their variances, denoted as ${\hat{I}}_{j}{\left({\hat{\beta }}_{j}\right)}^{-1}$. The second step involves calculating the false positive rate using the formula $q_{n}=\frac{f}{p_{n}}$, where $p_{n}$ refers to the total number of covariates and the parameter *f* is chosen based on practical needs. The threshold $\gamma_{n}$ is then set as $\gamma_{n}=\Phi^{-1}\left(1-\frac{q_{n}}{2}\right)$, with $\Phi^{-1}\left(x\right)$ representing the inverse cumulative distribution function of the standard normal distribution. In the final step, covariates are retained if they satisfy the condition $\sqrt{{\hat{I}}_{j}\left({\hat{\beta }}_{j}\right)}\left|{\hat{\beta }}_{j}\right|\geq \gamma_{n}$.

#### 2.3.2. Penalized Regression and Information-Theoretic Methods

The objective function of penalized Cox regression method can be expressed as follows:

(3) $$Q\left(\beta \right)=\ell \left(\beta \right)-\sum_{j=1}^{p}P_{\lambda }\left(\beta_{j}\right),$$

where ℓ(β) is the log-partial likelihood function as specified in Equation (2). Maximizing this objective function is key to identifying the most relevant features within the dataset. This approach employs various regularization techniques, such as the Lasso and SCAD, to achieve feature selection. Lasso imposes an L1 penalty on the coefficients to encourage sparsity, thereby shrinking less important coefficients towards zero [9]. On the other hand, SCAD introduces a non-convex penalty that aims to address the bias inherent in Lasso, especially for larger coefficient estimates [10]. The selection of penalized regression models is further supported by recent studies that demonstrate their superior performance in high-dimensional, small-sample settings common in RNA-seq and similar datasets, where methods like elastic net have shown optimal predictive accuracy [24].

In addition, we utilize information gain as one of the feature selection methods grounded in information theory [11]. For a given feature $X_{j}$, the information gain $IG(X_{j})$ is calculated as

(4) $$IG\left(X_{j}\right)=H\left(Y\right)-H\left(Y|X_{j}\right),$$

where $H(Y|X_{j})$ denotes the conditional entropy of *Y* given feature $X_{j}$, and H(Y) represents the entropy of the target feature *Y*. By emphasizing features with the greatest information gain, we can concentrate on those that effectively decrease ambiguity regarding the target feature, thereby boosting the model’s predictive accuracy overall.

### 2.4. Machine Learning-Based Methods

The survival tree method, based on the classification and regression tree (CART) framework, is tailored to accommodate survival data with censored observations. This method creates a binary decision tree using recursive partitioning, beginning at the root node. At each step, the algorithm divides the data based on criteria that aim to maximize differences in survival outcomes between the resulting groups. This splitting process is guided by a measure of statistical significance that ensures the most informative partitioning. The recursive partitioning continues until a specified stopping condition is reached, resulting in distinct subgroups with varied survival characteristics. The final survival tree structure provides a clear and interpretable representation of the data, allowing users to easily visualize the relationships between covariates and survival outcomes. This graphical format not only simplifies the understanding of the method’s results, but also highlights potential interactions and non-linear effects in the survival data. As a result, survival trees offer a powerful tool for uncovering complex patterns in survival analyses.

Random survival forests extend the standard random forest technique to effectively analyze right-censored survival data by constructing an ensemble of survival trees and combining their cumulative hazard estimates [16,17]. The process begins with generating multiple bootstrap samples, each containing about 63% of the original dataset, while the remaining data are used as out-of-bag (OOB) samples for error estimation. For each bootstrap sample, a survival tree is constructed by randomly selecting a subset of features at each node and determining the best split to maximize differences in survival outcomes between child nodes. The trees are grown to a specified depth, ensuring that each terminal node contains a minimum number of observations. Each individual tree is cumulative hazard function is calculated, and these are then averaged across all trees in the ensemble to obtain the overall cumulative hazard estimate. The OOB data are subsequently employed to assess the prediction error, providing a robust evaluation of the method’s performance. Throughout this process, the splitting criteria are designed to account for both survival times and censoring, allowing the method to effectively capture complex relationships in survival data.

### 2.5. Performance Metrics

To assess the performance of survival analysis methods in this study, we employ a range of metrics: the time-dependent receiver operating characteristic (ROC) curve, area under the curve (AUC), concordance index (C-index), specificity, sensitivity, negative predictive value (NPV), and positive predictive value (PPV). The time-dependent ROC curve, specifically adjusted for survival analysis, evaluates prediction accuracy at various time intervals by calculating sensitivity and specificity.

Specificity at a specified time point *t* is computed as

(5) $$\mathrm{SPE}\left(t\right)=\frac{\mathrm{TN}(t)}{\mathrm{FP}(t)+\mathrm{TN}(t)},$$

where TN(t) represents true negatives, and FP(t) stands for false positives. Sensitivity, or the true positive rate, calculates the proportion of correctly predicted cases,

(6) $$\mathrm{SEN}\left(t\right)=\frac{\mathrm{TP}(t)}{\mathrm{FN}(t)+\mathrm{TP}(t)},$$

where TP(t) indicates true positives, and FN(t) denotes false negatives. NPV measures the percentage of true negatives within all negative predictions,

(7) $$\mathrm{NPV}\left(t\right)=\frac{\mathrm{TN}(t)}{\mathrm{TN}(t)+\mathrm{FN}(t)}.$$

Similarly, PPV calculates the percentage of true positives among all positive predictions,

(8) $$\mathrm{PPV}\left(t\right)=\frac{\mathrm{TP}(t)}{\mathrm{TP}(t)+\mathrm{FP}(t)}.$$

These metrics allow for the generation of the ROC curve, with AUC providing a measure of predictive accuracy. Using the timeROC package in R, we create the ROC curve and calculate the AUC for survival data, which accounts for data censoring [25]. Additionally, the C-index evaluates concordance between predicted survival probabilities and observed outcomes,

(9) $$C_{\mathrm{index}}=\frac{\sum_{i,j\in \Omega }\left(I\left\{{\hat{s}}_{i}<{\hat{s}}_{j}\right\}+0.5\times I\left\{{\hat{s}}_{i}={\hat{s}}_{j}\right\}\right)}{\left|\Omega \right|},$$

where *I* denotes the indicator function, and Ω represents all relevant patient pairs. The C-index provides insight into the model’s ranking accuracy for survival times, serving as a measure of discriminative capability. This set of metrics underscores the utility of survival analysis techniques in clinical applications.

## 3. Results

### 3.1. Identification of Significant Genetic Markers

We start our analysis of the ultra-high dimensional lung adenocarcinoma (LUAD) RNA-seq data by utilizing the principled sure independence screening (PSIS) method, following the guidelines outlined by Zhao and Li [8]. This method is used to effectively reduce the number of features in our dataset, narrowing it down to 61 gene features. In accordance with Zhao and Li’s recommendations [8], we set the parameter f=1 to manage the false positive rate, optimizing our selection of relevant predictors. Following this, we refine our analysis using additional feature selection methods, such as Lasso and SCAD, along with information gain (IG) [9,10,11]. These approaches, paired with PSIS, are designated as PSIS-Lasso, PSIS-SCAD, and PSIS-IG, respectively. This comprehensive strategy not only improves our ability to select significant gene features, but also ensures a more precise evaluation of the dataset’s most relevant predictors.

The feature selection results in Table 2 highlight the distinct and overlapping capabilities of the three methods (PSIS-Lasso, PSIS-SCAD, and PSIS-IG) in identifying key features associated with the study’s outcome. Each method selects a unique set of genes, with PSIS-Lasso identifying 15 genes, PSIS-SCAD selecting 14 genes, and PSIS-IG highlighting 9 genes, reflecting their different selection criteria and strengths. PSIS-Lasso, known for its ability to handle high-dimensional data by promoting sparsity in feature selection, uniquely identifies several genes such as *OPN3*, *RHOV*, and *CDX2*. These genes are not picked by PSIS-SCAD or PSIS-IG, which may imply that Lasso’s shrinkage properties allow it to capture features with subtle effects that might be overlooked by non-convex or information-theoretic approaches. This characteristic highlights Lasso’s sensitivity to a broader range of predictive patterns in the data. PSIS-SCAD, on the other hand, identifies unique genes like *FAM83A*, *UNC5D*, and *MT2P1*. SCAD’s non-convex penalty is specifically designed for addressing the limitations of Lasso, such as the estimation bias with larger coefficients [10]. This feature of SCAD enables it to retain more relevant features when dealing with strongly predictive variables, suggesting that these unique genes might have a higher impact or stronger associations with the outcome that are not emphasized by Lasso’s penalty structure. PSIS-IG’s selection is more conservative, identifying only nine genes, including unique candidates like *ARNTL2*, *BIRC3*, and *VEGFC*. The focus of IG on reducing entropy and quantifying the amount of information gained by each feature suggests that these genes have a specific relevance in explaining the variability of the survival outcome. The selection of *VEGFC* and other unique genes by IG highlights its strength in pinpointing features that directly contribute to the reduction in uncertainty, which is crucial in understanding the most informative predictors in the dataset.

The fact that each method selects a combination of both overlapping and unique features illustrates the complementary nature of these approaches. The method-specific selections suggest that different techniques capture distinct aspects of the data’s structure. This multi-faceted approach to feature selection not only enhances the robustness of the findings, but also broadens the analytical perspective, potentially revealing a more comprehensive set of biomarkers. Notably, *DKK1* and *TNS4* are consistently chosen by all three methods: PSIS-Lasso, PSIS-SCAD, and PSIS-IG. This agreement suggests a high level of robustness for these genes as significant predictors, indicating their potential as core biomarkers in the context of the study. The consistent selection of these genes by diverse methods underscores their possible biological significance and strengthens their candidacy for further investigation in survival analysis.

**Figure 3:**
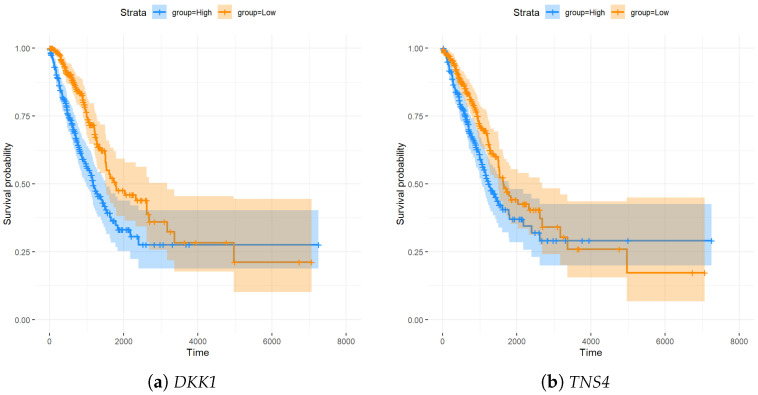
Kaplan–Meier analysis illustrating survival outcomes for *DKK1* and *TNS4* across high- and low-expression groups.

Figure 3 illustrates Kaplan–Meier survival curves for *DKK1* and *TNS4*, showing marked survival outcome disparities between groups with high and low gene expression levels. For *DKK1*, the survival probability in the high-expression group is significantly lower than that of the low-expression group. This suggests that high *DKK1* expression may be associated with poorer patient prognosis, underscoring its potential utility as a predictive marker in lung adenocarcinoma. Similarly, the Kaplan–Meier curve for *TNS4* shows a clear distinction in survival outcomes between groups with high and low expression levels. The data indicate that patients with higher *TNS4* expression levels tend to have a lower survival probability over time compared to those with lower expression. Although the effect is not as pronounced as that of *DKK1*, the association remains statistically significant, suggesting that *TNS4* may also serve as a valuable marker in predicting survival outcomes in this context. The clear separation in survival curves for both genes emphasizes their potential clinical relevance. The consistency in their identification across different feature selection methods further supports their robustness as biomarkers. These findings underscore the importance of integrating *DKK1* and *TNS4* into prognostic models to better stratify patients based on their risk and improve personalized treatment strategies for lung adenocarcinoma.

Overall, the use of PSIS-Lasso, PSIS-SCAD, and PSIS-IG together provides a well-rounded feature selection process that balances sensitivity to a wide range of predictors with the ability to zero in on the most informative genes. This strategy ensures that critical biomarkers are identified while also uncovering subtle, yet significant, genetic influences on survival, ultimately contributing to a deeper understanding of the biological factors driving the study’s outcomes.

**Figure 4:**
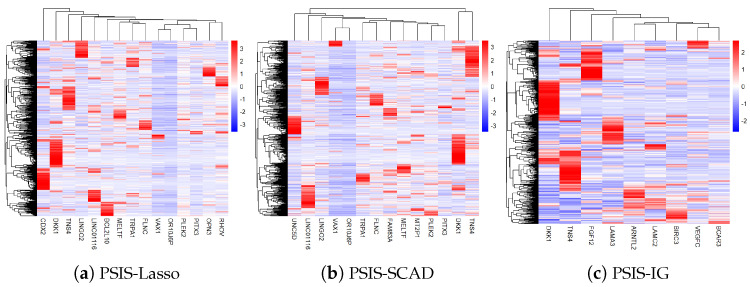
Gene heatmaps of features selected by PSIS-Lasso (**a**), PSIS-SCAD (**b**), and PSIS-IG (**c**).

The gene heatmaps in Figure 4 display the expression patterns of features selected by PSIS-Lasso, PSIS-SCAD, and PSIS-IG, revealing the distinct and overlapping profiles identified by each method. In the PSIS-Lasso heatmap (Figure 4a), genes such as *DKK1*, *TNS4*, and *OPN3* show prominent expression differences, reflecting Lasso’s ability to highlight a wide range of predictive features due to its sparse regularization. PSIS-SCAD (Figure 4b) captures unique expression patterns for genes like *FAM83A*, *UNC5D*, and *MT2P1*, indicating SCAD’s strength in identifying features with stronger predictive signals, thanks to its non-convex penalty that reduces bias in large coefficients. Meanwhile, the PSIS-IG heatmap (Figure 4c) emphasizes genes such as *ARNTL2*, *VEGFC*, and *BIRC3*, showcasing IG’s focus on selecting features that help reduce uncertainty in the target feature. These heatmaps highlight the complementary nature of the methods, with some genes like *DKK1* and *TNS4* consistently identified across all approaches, underscoring their robustness as biomarkers. By combining insights from PSIS-Lasso, PSIS-SCAD, and PSIS-IG, we achieve a more comprehensive view of gene expression patterns, enhancing our understanding of key biological processes in lung adenocarcinoma.

**Figure 5:**
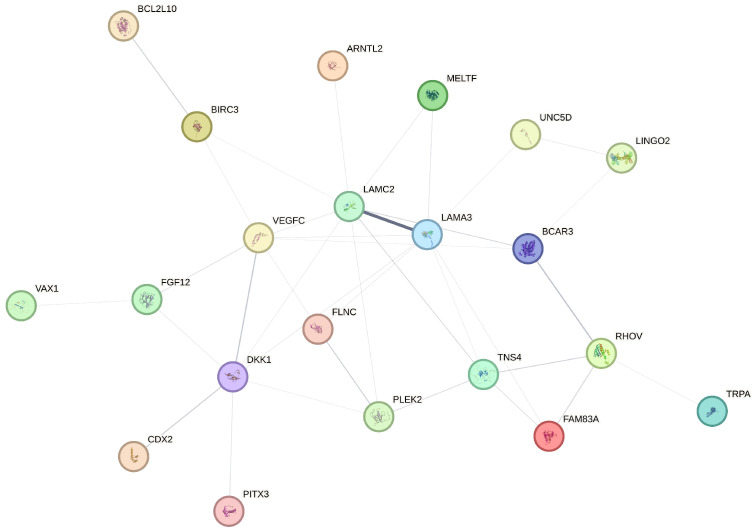
Network visualization of protein–protein interactions among the 21 key genes.

The STRING database provides an essential platform for building protein–protein interaction networks, merging data from various sources to reveal associations among proteins [26]. In our research, we utilize STRING to construct a network for the 25 genes identified via our feature selection approaches. To ensure that the visualization focuses on biologically significant interactions, we set a confidence threshold of 0.15, including interactions backed by robust evidence. Due to this threshold, four genes, specifically *BCL2L10*, *OR10J6P*, *CDX2*, and *MT2P1*, do not meet the minimum interaction criteria and are thus omitted from the final network, resulting in a streamlined network of 21 genes, as depicted in Figure 5. In this network, each gene is represented as a node, with edges connecting them to indicate protein–protein interactions, where the edge thickness reflects the interaction confidence level.

The protein–protein interaction (PPI) network illustrated in Figure 5 showcases the intricate relationships among the 21 selected genes, forming a complex network of interlinked nodes that suggest potential cooperative roles. Key central nodes, such as *LAMC2*, *LAMA3*, and *VEGFC*, exhibit numerous connections with other proteins, underscoring their function as primary interaction hubs. This central positioning indicates that these genes may play an essential role in modulating molecular pathways critical to lung adenocarcinoma (LUAD). Furthermore, genes like *BCAR3*, *DKK1*, and *TNS4* are linked to multiple proteins, highlighting their significance within the study context and suggesting that they may participate in coordinated pathways associated with LUAD progression and treatment response. In contrast, genes with fewer connections, such as *CDX2*, *RHOV*, and *FAM83A*, may fulfill more specialized functions that warrant further investigation to understand their specific roles in disease mechanisms. This network analysis not only underscores the biological relevance of each gene, but also supports the robustness of the selected biomarkers by demonstrating their involvement in established protein interactions. These network interactions imply that the selected genes potentially work together within cellular systems, highlighting both primary nodes with wide-reaching influence and peripheral nodes that might participate in more specific pathways. The insights derived from this PPI network can direct future research toward targeted therapeutic approaches or biomarker development for LUAD, using these genes as focal points for exploring their molecular roles and interactions further.

### 3.2. Performance Evaluation of Cox Model and Machine Learning-Based Methods

Following the feature selection process, which combines PSIS with Lasso, SCAD, and Information Gain, we proceed to implement survival analysis methods: Cox proportional hazards model (Cox), survival tree (ST), and random survival forest (RSF). To evaluate the predictive accuracy of these methods, we employ a 10-fold cross-validation technique. It involves splitting the genetic dataset into ten unique subsets. For each iteration, nine subsets are used to train the model, while the other subset serves as the test set. This cycle is repeated ten times to ensure thorough validation. In each round, models are trained on the designated training subset and subsequently assessed on the test subset. Performance is evaluated based on key metrics, including receiver operating characteristic (ROC) curve, area under the curve (AUC), concordance index (C-index), sensitivity, specificity, negative predictive value (NPV), and positive predictive value (PPV).

**Figure 6:**
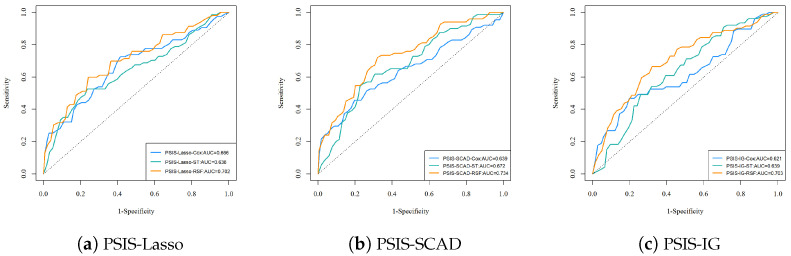
Comparison of ROC curves and AUC metrics for each survival analysis method across feature selection methods: (**a**) PSIS-Lasso, (**b**) PSIS-SCAD, and (**c**) PSIS-IG, showing the effectiveness of Cox, ST, and RSF methods.

The AUC values and ROC curves in Figure 6 offer a clear comparison of the performance of the Cox model, ST, and RSF across the feature selection methods: PSIS-Lasso, PSIS-SCAD, and PSIS-IG. For the PSIS-Lasso feature selection, RSF achieves the highest AUC value of 0.702, indicating a relatively strong capability in distinguishing between survival outcomes compared to the Cox model with an AUC of 0.666 and ST with 0.638. This trend persists with PSIS-SCAD, where RSF outperforms other methods with an AUC of 0.734, followed by ST at 0.672 and Cox at 0.639. For PSIS-IG, RSF again leads with an AUC of 0.703, demonstrating its robustness in survival prediction, while ST and Cox lag slightly behind with AUC values of 0.639 and 0.621, respectively. These results consistently position RSF as the most effective survival analysis method among the three, particularly when used in conjunction with different feature selection techniques, reflecting its strength in handling the complexities of high-dimensional genetic data.

The detailed performance metrics in Table 3 further emphasize the advantages of RSF across several key criteria. Sensitivity, which measures the true positive rate, is notably higher for RSF in all scenarios, with values like 0.754 for PSIS-Lasso, 0.760 for PSIS-SCAD, and reaching 0.880 for PSIS-IG, indicating that RSF is particularly adept at correctly identifying patients at risk. Similarly, RSF consistently shows strong negative predictive values (NPV), such as 0.924 with PSIS-Lasso and 0.946 with PSIS-IG, suggesting that it effectively minimizes the likelihood of false negatives. These high sensitivity and NPV scores underline RSF’s reliability in ensuring that individuals predicted as low-risk are indeed less likely to experience adverse outcomes. In terms of specificity, which evaluates the true negative rate, RSF exhibits relatively moderate values compared to its strong sensitivity, indicating some trade-offs in its ability to accurately classify patients who are not at risk. For instance, RSF achieves specificity values of 0.424 for PSIS-Lasso, 0.450 for PSIS-SCAD, and 0.357 for PSIS-IG, highlighting room for improvement in its performance on true negative predictions. Despite this, RSF’s C-index scores, which reflect the concordance between predicted risks and actual survival times, are consistently higher than those of Cox and ST methods, pointing to its superior ability to rank patients according to their risk levels accurately. However, the lower positive predictive value (PPV) across all feature selection methods, such as 0.146 for PSIS-Lasso and 0.160 for PSIS-IG, suggests that while RSF is effective at identifying those who are at risk, it is less precise in predicting true positive cases.

**Figure 7:**
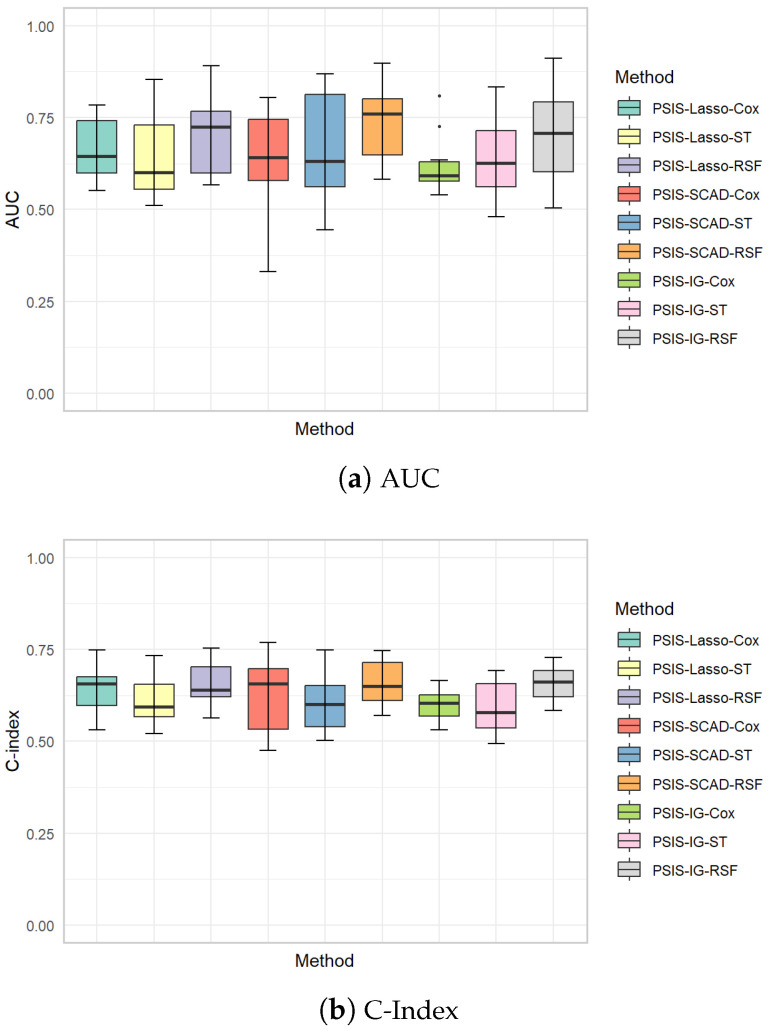
Boxplots illustrating AUC (**a**) and C-index (**b**) values for various survival analysis methods applied to different feature selection methods.

The boxplots in Figure 7 provide a comparative view of the AUC and C-index distributions for the survival analysis methods applied to different feature selection methods. According to the AUC boxplot, it is evident that the RSF generally show higher median AUC values across all feature selection methods, with less variation in their performance compared to the Cox and ST methods. This consistency in higher AUC values reflects RSF’s strong capability to distinguish high-risk patients from low-risk ones across various datasets, emphasizing its effectiveness in managing complex survival data. Similarly, the C-index boxplot illustrates that RSF tends to outperform the Cox and ST methods in terms of ranking accuracy, as indicated by its higher median values and relatively narrow interquartile range. The results suggest that RSF provides more reliable and stable predictions of survival outcomes across varying conditions, reinforcing its suitability for clinical applications where precise risk stratification is crucial. While the Cox and ST methods show more variability in both AUC and C-index values, their performance is still competitive in certain scenarios, indicating that they may still be valuable in contexts where simpler methods are preferred or computational efficiency is a priority.

In summary, random survival forests consistently outperform the Cox and survival tree methods in predicting survival outcomes across all feature selection techniques, as shown by higher AUC, C-index, and sensitivity values. While Cox and ST remain useful in scenarios requiring simpler methods, RSF’s robust performance in capturing complex patterns makes it the most effective approach for risk stratification in clinical settings.

## 4. Discussion

In this study, we applied an integrated approach combining principled sure independence screening (PSIS) with penalized regression techniques and information gain to perform feature selection on ultra-high-dimensional lung adenocarcinoma (LUAD) RNA-seq data. Our multi-step strategy successfully reduced the dimensionality of the dataset, ultimately highlighting 61 gene features that serve as potential biomarkers for LUAD. One of the most significant outcomes of this investigation was the identification of *DKK1* and *TNS4* as consistent biomarkers across all three feature selection techniques: PSIS-Lasso, PSIS-SCAD, and PSIS-IG. The Kaplan–Meier method showed a strong association between high expression levels of these genes and reduced patient survival, suggesting their potential roles as critical prognostic indicators in LUAD. This consistency across multiple methodologies strengthens the reliability of *DKK1* and *TNS4* as biomarkers, underscoring their potential utility in clinical applications. These findings are consistent with prior studies showing that elevated *DKK1* levels are associated with poor outcomes in several cancers, including lung cancer, where it is believed to impact tumor progression and metastasis through its role in the Wnt signaling pathway. Similarly, *TNS4* has been implicated in cell migration and invasion, which are key processes in cancer progression, further supporting its candidacy as a prognostic marker. However, we recognize the limitation of our study in not determining the optimal threshold values for these biomarkers and in not performing multiple testing adjustments, which are essential steps to enhance clinical applicability and validate biomarker efficacy across different cohorts [27]. Future work could focus on addressing these aspects to further strengthen the robustness of our findings.

The majority of genes identified in Table 2 have been confirmed in relevant studies to be associated with LUAD carcinogenesis. Overexpression of *RHOV* promoted proliferation, migration, and invasion of LUAD cells, while knockdown of *RHOV* inhibited these biological behaviors [28]. Zhou et al. [29] utilized a graph-based learning dimensionality reduction analysis to identify *PLEK2* as an antigen related to LUAD, which was highly correlated with immune infiltrating cells and poor clinical outcomes. Zhang et al. [30] experimentally validated that inhibition of *TRPA1* expression could enhance the sensitivity of lung cancer cells to radiation, potentially providing new targets for the combined treatment of lung cancer with radiotherapy and immunotherapy. Li et al. [31] found that *PITX3* was one of the key gene features for analyzing LUAD prognosis. Yao et al. [32] confirmed that the upregulation of DDK1 could inactivate the Wnt/β-catenin pathway, thereby blocking the progression of LUAD carcinogenesis. Misono et al. [33] discovered abnormal expression of *TNS4* in clinical specimens of LUAD, which increased the invasiveness of LUAD cells. A study revealed for the first time that *LINC01116* drove oncogenic activity in LUAD by scaffolding essential transcription factors to the ribosomal DNA promoter, thereby enhancing Pol I transcription [34]. In cellular experiments, *MELTF* was shown to promote the malignant progression of LUAD cells [35]. Zhang et al. [36] found that *FAM83A* was overexpressed in LUAD, and its overexpression served as an independent factor for poor prognosis in LUAD patients. Overexpression of *ARNTL2* conferred a poor prognosis to LUAD patients [37]. *LAMA3* was a gene positively correlated with drug resistance in LUAD [38].

The subsequent survival analysis using methods like Cox model, survival tree (ST), and random survival forest (RSF) provided valuable insights into the prognostic significance of these genetic markers. The Cox model, as a traditional survival analysis tool, demonstrated good performance in cancer prognostic prediction. In the study by Lee and Lim [39], the Cox model was utilized to predict pancreatic ductal adenocarcinoma (PDAC) using only genetic data. Survival trees not only served to predict cancer, but also acted as exploratory tools, revealing new insights into gene expression profiles. Berrar et al. [40] utilized survival trees to identify the genes netrin receptor neogenin and the Ras/Rho kinase regulator diacylglycerol kinase α as key factors influencing lung adenocarcinoma. Ishwaran et al. [41] discussed the application of RSF in the analysis of high-dimensional survival data and emphasized its effectiveness and superiority in genomic research.

Our comparison of survival analysis methods revealed that RSF consistently surpassed both the Cox and ST models in predictive accuracy, as indicated by metrics such as the concordance index (C-index), area under the ROC curve (AUC), and sensitivity. The superior performance of RSF in these analyses highlights its robustness in managing complex, non-linear relationships within high-dimensional data, making it a highly suitable method for risk stratification in LUAD patients. RSF’s capacity to manage covariate interactions and address the specific censoring structure in survival data makes it a valuable tool for clinical decision-making, particularly with complex genetic datasets.

Despite these strengths, some limitations should be considered. While RSF achieved excellent sensitivity and negative predictive value (NPV), its specificity and positive predictive value (PPV) were relatively lower by comparison. This trade-off suggests that while RSF is highly effective at identifying individuals at risk, it may struggle with accurately predicting patients who will not experience adverse outcomes. Such limitations highlight the need for complementary approaches that can enhance the specificity of RSF-based predictions, perhaps through combining RSF with simpler methods like the Cox regression when the emphasis is on minimizing false positives.

The protein–protein interaction (PPI) network analysis further enriched our understanding of the biological relevance of the selected genes. By focusing on genes that interact most robustly with others in the network, such as *LAMC2*, *LAMA3*, and *VEGFC*, we identified key hubs that may play central roles in LUAD pathophysiology. These hub genes could be critical in maintaining cellular communication and signaling pathways that drive tumor progression. The exclusion of genes like *BCL2L10*, *OR10J6P*, *CDX2*, and *MT2P1* from the PPI network due to lack of interaction data may also warrant further investigation into their specific molecular functions or how their roles might be context-dependent within the tumor microenvironment.

Additionally, the gene expression heatmaps created for the PSIS-Lasso, PSIS-SCAD, and PSIS-IG methods revealed distinct expression patterns, emphasizing each method’s unique ability to capture relevant biological signals. For example, PSIS-Lasso’s emphasis on sparsity helped identify subtle gene expressions, whereas PSIS-SCAD’s non-convex penalties allowed for capturing genes with stronger associations to the outcome. IG’s information-theoretic focus on entropy reduction provided insights into the most informative features directly linked to survival outcomes. The diversity in these results suggests that a multi-pronged approach to feature selection may be most beneficial, as it provides a more holistic view of gene activity in cancer biology.

Overall, our findings underscore the importance of using a combination of advanced feature selection techniques and robust survival analysis methods in genomic research. By employing PSIS, Lasso, SCAD, and IG in concert with machine learning approaches like RSF, we not only enhance our understanding of genetic drivers in LUAD, but also pave the way for more precise and personalized medical treatments. This integrative approach represents a significant step toward leveraging molecular data to improve patient outcomes and advancing the field of precision oncology.

## 5. Conclusions

In this work, we developed a robust approach for identifying key genetic markers in lung adenocarcinoma by integrating principled sure independence screening with penalized regression techniques and information gain. By applying PSIS-Lasso, PSIS-SCAD, and PSIS-IG methods, we successfully highlighted *DKK1* and *TNS4* as consistent predictors of patient survival, underscoring their potential as core biomarkers for LUAD prognosis. These findings are strengthened by Kaplan–Meier method, which revealed a significant association between high expression levels of these genes and poor survival outcomes. Our comparative analysis of survival methods demonstrated that random survival forests deliver superior predictive performance over traditional methods like the Cox model and survival trees. RSF’s ability to handle complex interactions in high-dimensional datasets makes it an ideal tool for clinical risk stratification and decision-making in oncology. The construction of the protein–protein interaction network provided additional insight into the functional roles of the selected genes, pinpointing *LAMC2*, *LAMA3*, and *VEGFC* as central nodes that could play crucial roles in tumor development and progression. These network findings suggest potential avenues for therapeutic intervention and highlight the importance of understanding gene interactions within molecular pathways.

In conclusion, our study’s integrated methodology not only refines the identification of crucial biomarkers in LUAD, but also enhances the precision of survival predictions. These findings lay the groundwork for crafting personalized treatment strategies, underlining the importance of molecular profiling in enhancing patient outcomes. Future studies should prioritize the validation of these results in independent cohorts to confirm their clinical applicability and explore the translational potential of these biomarkers in tailored treatment approaches. Additionally, expanding this work to include multi-omics data or external validation datasets would further strengthen the robustness and generalizability of our findings, potentially revealing more comprehensive insights into LUAD pathogenesis and treatment responses.

## Figures and Tables

**Table 1:** Descriptive statistics for the four genes, showing the minimum (Min), first quartile (Q1), median, mean, third quartile (Q3), and maximum (Max) expression values.

	*MT-CO1*	*AL356310.1*	*RPL21P44*	*AL162151.3*
Min	10,612	0.000	0.000	0.000
Q1	241,713	0.000	1.000	0.000
Median	390,805	2.000	3.000	0.000
Mean	482,273	3.427	3.427	0.480
Q3	630,284	4.000	4.000	1.000
Max	3,996,161	61.000	27.000	6.000

**Table 2:** Selected features by PSIS-Lasso, PSIS-SCAD, and PSIS-IG.

Feature	PSIS-Lasso	PSIS-SCAD	PSIS-IG
*OPN3*	✓		
*PLEK2*	✓	✓	
*RHOV*	✓		
*TRPA1*	✓	✓	
*PITX3*	✓	✓	
*DKK1*	✓	✓	✓
*FLNC*	✓	✓	
*TNS4*	✓	✓	✓
*BCL2L10*	✓		
*VAX1*	✓	✓	
*OR10J6P*	✓	✓	
*LINC01116*	✓	✓	
*MELTF*	✓	✓	
*CDX2*	✓		
*LINGO2*	✓	✓	
*FAM83A*		✓	
*UNC5D*		✓	
*MT2P1*		✓	
*ARNTL2*			✓
*BIRC3*			✓
*LAMC2*			✓
*FGF12*			✓
*VEGFC*			✓
*LAMA3*			✓
*BCAR3*			✓
**Count**	15	14	9

**Table 3:** Evaluation metrics for various survival analysis methods applied with different feature selection methods.

Metrics	PSIS-Lasso			PSIS-SCAD			PSIS-IG		
	**Cox**	**ST**	**RSF**	**Cox**	**ST**	**RSF**	**Cox**	**ST**	**RSF**
AUC	0.666	0.638	0.702	0.639	0.672	0.734	0.621	0.639	0.703
C-index	0.639	0.613	0.656	0.631	0.604	0.660	0.599	0.593	0.657
Sensitivity	0.803	0.824	0.754	0.766	0.738	0.760	0.813	0.625	0.880
Specificity	0.335	0.302	0.424	0.345	0.415	0.450	0.260	0.539	0.357
NPV	0.902	0.917	0.924	0.867	0.929	0.924	0.925	0.921	0.946
PPV	0.139	0.143	0.146	0.147	0.132	0.161	0.137	0.139	0.160

## Data Availability

The data presented in the study are available in the Cancer Genome Atlas database at https://portal.gdc.cancer.gov, accessed on 6 February 2024.

## Abbreviations

The following abbreviations are used in this manuscript:

LUAD	Lung Adenocarcinoma
TCGA	The Cancer Genome Atlas
GDC	Genomic Data Commons
PSIS	Principled Sure Independence Screening
Lasso	Least Absolute Shrinkage and Selection Operator
SCAD	Smoothly Clipped Absolute Deviation
IG	Information Gain
ST	Survival Tree
RSF	Random Survival Forest
ROC	Receiver Operating Characteristic
AUC	Area Under the Curve
C-index	Concordance Index
